# Spontaneous transomental internal hernia causing small bowel obstruction in an elderly patient with a virgin abdomen: a case report

**DOI:** 10.1093/jscr/rjag835

**Published:** 2026-09-29

**Authors:** Zafer Goren, Yasar Kalip

**Affiliations:** Kayseri City Hospital, General Surgery Clinic, Kayseri, Turkey; Kayseri City Hospital, General Surgery Clinic, Kayseri, Turkey

**Keywords:** transomental hernia, internal hernia, small bowel obstruction, ileus, closed-loop obstruction, case report

## Abstract

Internal hernias are a rare cause of small bowel obstruction (SBO), accounting for ~0.5%–5% of cases; transomental hernia represents the rarest subtype, comprising only 1%–4% of internal hernias. We report a 72-year-old man with hypertension but no prior abdominal surgery or trauma who presented with abdominal pain, nausea, and vomiting. Laboratory tests showed leukocytosis; abdominal radiography demonstrated air-fluid levels. Contrast-enhanced computed tomography revealed a collapsed colon, a distinct transition zone between dilated proximal and collapsed distal small bowel loops, and localized mesenteric fat stranding, suggesting internal hernia. After 48 h without improvement on conservative management, exploratory laparotomy revealed a 3-cm circular defect in the greater omentum with a herniated, viable small bowel loop causing closed-loop obstruction. The loop was reduced and the defective omental segment resected without bowel resection. The patient recovered uneventfully and was discharged on postoperative day 3.

## Introduction

Internal hernia is defined as protrusion of a viscus, most commonly a small bowel loop, through a congenital or acquired defect in the peritoneum, mesentery, or omentum, without formation of a hernial sac [1, 2]. It accounts for ~0.5%–5% of small bowel obstruction (SBO) cases [2–4]. According to Meyers’ classification, paraduodenal (53%) and pericaecal (13%) hernias are most common, followed by foramen of Winslow (8%), transmesenteric/transmesocolic (8%), and pelvic/intersigmoid hernias (6–7%); transomental hernia (TOH) is the rarest subtype, comprising only 1–4% of internal hernias [3, 4].

Omental defects causing TOH may be congenital or acquired. Acquired defects are most often related to prior abdominal surgery—classically Roux-en-Y gastric bypass—as well as trauma, prior intra-abdominal inflammation, or senile omental atrophy in elderly patients [4, 5]. Spontaneous TOH without any surgical or traumatic history is rare and shows a bimodal age distribution, affecting both paediatric patients and adults over 50 years of age [4]. Because the narrow, sharply defined omental orifice predisposes the herniated loop to a closed-loop configuration, TOH carries a substantial risk of strangulation; delayed diagnosis and treatment have been associated with mortality rates of up to 30%–45% [1, 2].

Clinical presentation is non-specific and mimics other causes of mechanical SBO, so preoperative diagnosis is rarely achieved [4, 6]. Computed tomography (CT) is the imaging modality of choice and may show clustered, abnormally located dilated bowel loops, a distinct transition point, and converging or engorged mesenteric vessels, although diagnosis is usually confirmed only at surgical exploration [4, 6]. Given its rarity, we report the clinical course, imaging findings, and surgical management of spontaneous TOH in an elderly patient without predisposing surgical or traumatic history, to contribute to the limited existing case literature.

## Case report

A 72-year-old man presented with abdominal pain, nausea, and vomiting. His medical history was notable only for hypertension treated with amlodipine 10 mg; he had no prior abdominal surgery or trauma. Physical examination showed no significant abdominal distension and no signs of peritonitis.

Laboratory investigation revealed leukocytosis (16 000/μL) with otherwise normal parameters. Erect abdominal radiography showed air-fluid levels. Contrast-enhanced abdominal CT demonstrated a completely collapsed colon, collapsed distal, and dilated proximal small bowel loops, and a distinct transition zone with localized mesenteric fat stranding (Figs 1–3), raising suspicion of internal (transomental) hernia.

**Figure 1:**
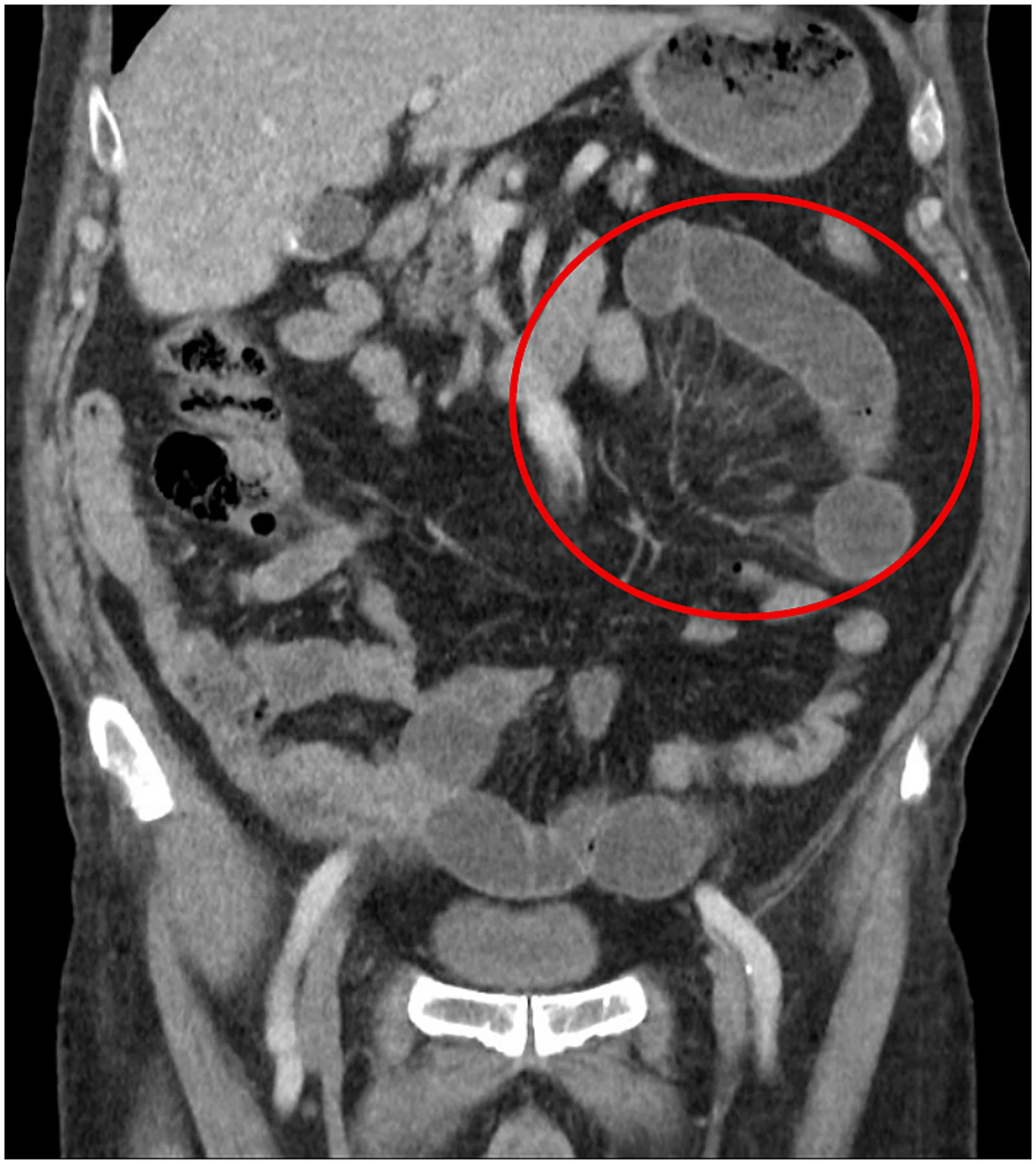
Coronal contrast-enhanced CT image showing increased mesenteric density in the herniated segment (circle).

**Figure 2:**
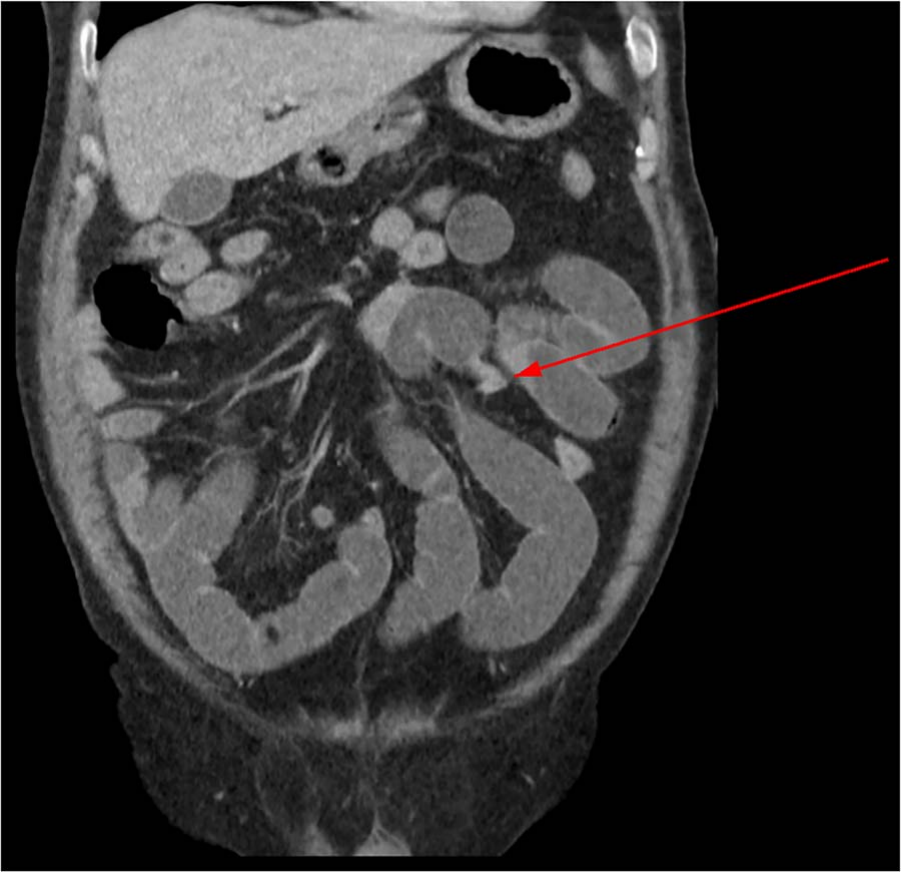
Coronal CT image showing findings of small bowel obstruction (arrow).

**Figure 3:**
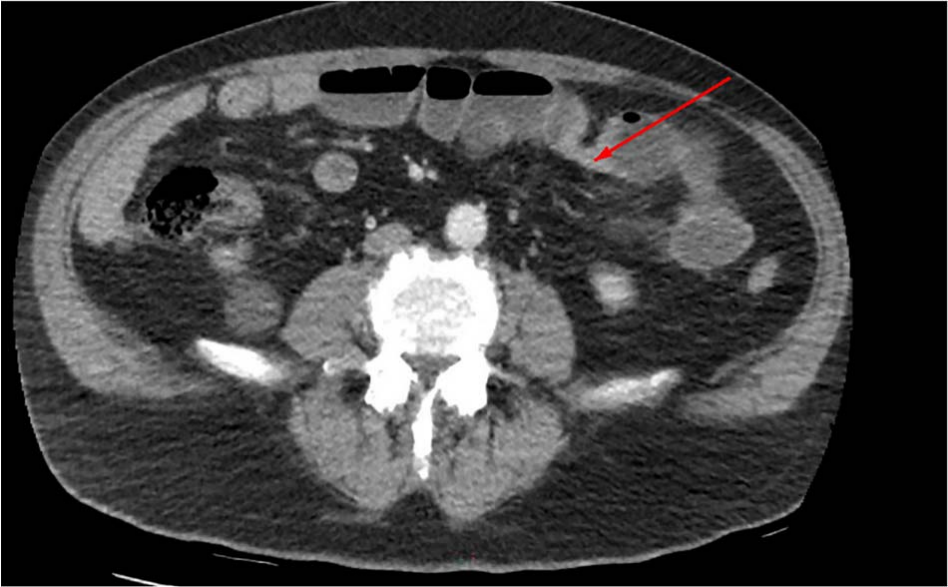
Axial CT image showing dilated and collapsed small bowel loops with the transition zone (arrow).

In the absence of peritonitis, the patient was initially managed conservatively under close observation. However, at 48 h, control radiographs showed persistent air-fluid levels without clinical improvement, prompting surgical exploration.

Through a midline supraumbilical laparotomy, a circular defect ~3 cm in diameter was identified in the greater omentum, with a small bowel loop and its mesentery herniated through it (Fig. 4). The proximal segment was dilated and the distal segment collapsed, confirming closed-loop obstruction. Mesenteric oedema and a ring of inflammatory change in the bowel wall at the defect level were noted, along with surrounding omental inflammation. After careful reduction of the herniated loop, the defective omental segment was resected. As there was no evidence of ischemic necrosis or perforation, bowel resection was not required; luminal patency was confirmed by gently milking bowel contents towards the ascending and transverse colon.

**Figure 4:**
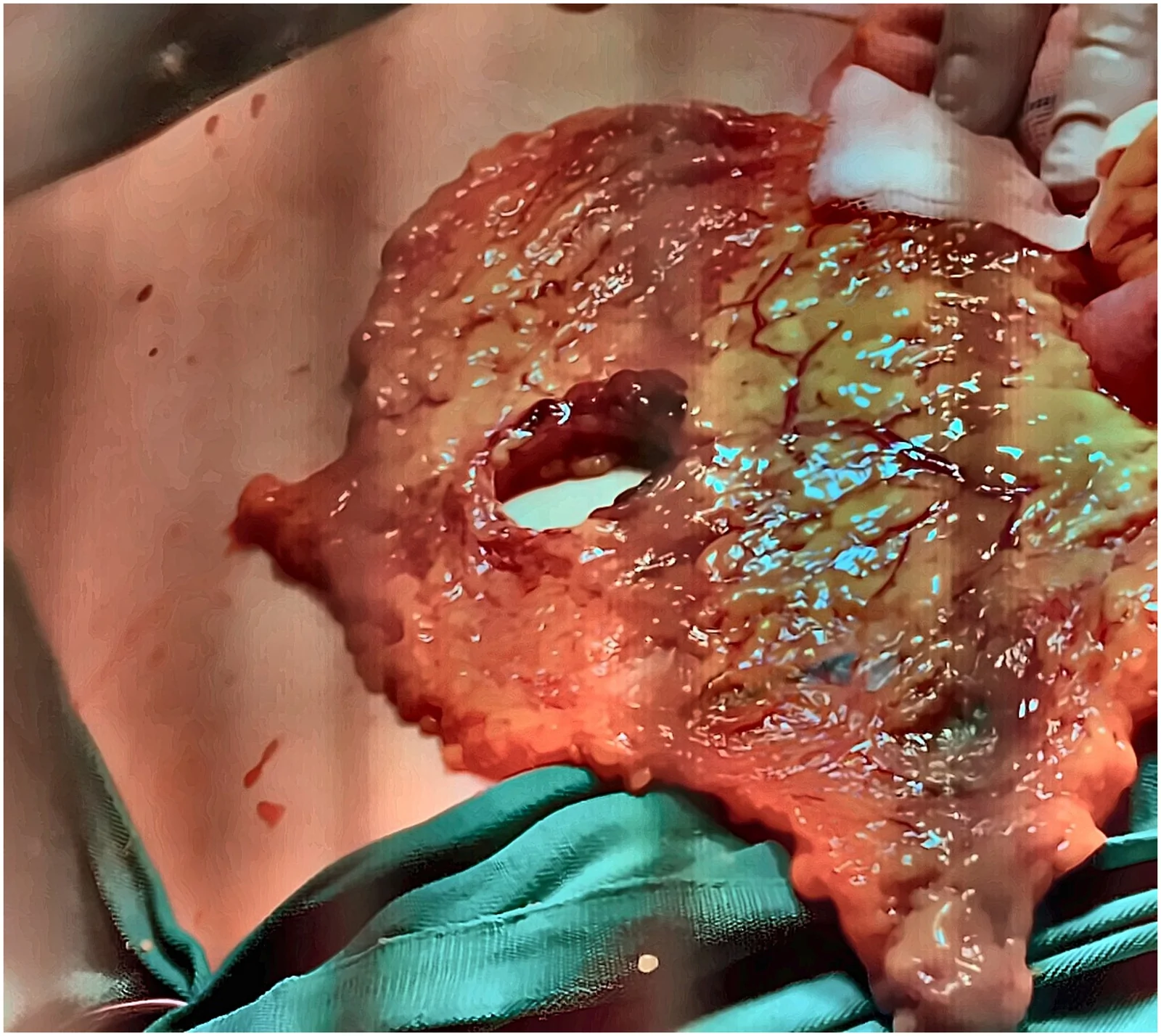
Intraoperative photograph showing the ~3-cm circular omental defect through which the small bowel loop was herniated.

Oral intake was resumed on postoperative day 1. The patient passed stool on day 2 and was discharged in stable condition on postoperative day 3.

## Discussion

TOH is the rarest internal hernia subtype, accounting for only 1%–4% of cases, with an even smaller share of SBO [1, 3, 4]. Yamaguchi classified transomental hernias into three anatomical types: Type A, through fused layers of the greater omentum (as in this case); Type B, via the omental bursa; and Type C, into the omental bursa [7]. Omental defects may be congenital or, more commonly in recent series, acquired through senile atrophy, prior inflammation, trauma, or previous abdominal surgery such as Roux-en-Y gastric bypass [4, 5]. Our patient’s absence of surgical or traumatic history and advanced age are consistent with the bimodal distribution described for spontaneous TOH, in which age-related omental atrophy is thought to predispose to defect formation [4].

Preoperative diagnosis of TOH is rarely achieved because its presentation—abdominal pain, nausea, vomiting, and variable distension—cannot be reliably distinguished from other causes of mechanical SBO [4, 6]. In our patient, CT showed increasingly recognized features suggestive of internal hernia: a distinct transition point, localized mesenteric fat stranding, and a proximal dilation–distal collapse pattern consistent with closed-loop obstruction. As in most reported cases, however, definitive diagnosis was confirmed only intraoperatively [4, 6]. This diagnostic difficulty is clinically important, since the narrow, sharply defined orifice of omental defects predisposes the herniated loop to vascular compromise, and delayed diagnosis has been associated with mortality of up to 30%–45% [1, 2].

Management is guided primarily by intraoperative assessment of bowel viability. When the herniated loop is viable, as in our case, reduction and excision of the defective omental segment suffice, avoiding bowel resection; segmental resection—sometimes extensive—is required if strangulation has progressed to ischemia or perforation [1, 5, 6]. Our patient’s favourable outcome, without bowel resection and discharge on postoperative day 3, underscores the benefit of timely surgical exploration when conservative management fails. Successful laparoscopic diagnosis and management of spontaneous TOH have also been reported [2, 4]; nonetheless, open surgery remains a reasonable approach, particularly when preoperative diagnosis is uncertain or clinical deterioration mandates urgent exploration, as in this case.

This report adds to the limited literature on spontaneous TOH and highlights two practical points: TOH should remain in the differential diagnosis of unexplained SBO even in patients without abdominal surgical or traumatic history, particularly in the elderly; and when clinical and radiological findings fail to improve, a low threshold for surgical exploration—rather than prolonged conservative management—is warranted to prevent progression to bowel strangulation.
